# Addressing health and ageing in the German national health monitoring system

**DOI:** 10.1186/1753-6561-7-S4-S12

**Published:** 2013-08-23

**Authors:** Christa Scheidt-Nave, Judith Fuchs, Yong Du, Ute Ellert, Anja Schienkiewitz, Markus Busch, Hildtraud Knopf, Beate Gaertner, Liv Bode

**Affiliations:** 1 Department of Epidemiology and Health Monitoring, Robert Koch Institute, Berlin, Germany

## 

Public health systems all over the world need to get adapted to the relatively new and unprecedented fact of longevity. Data from periodically repeated national health surveys can provide useful information to health researchers, health politicians, and the public by monitoring age-related morbidity, functional decline, and disability in relation to behavioural risk factors and context factors, including socioeconomic, political, and cultural factors.

In Germany, annually repeated national health interview surveys (German Health Update, GEDA) have been used to study patterns of multi- and comorbidity in the German adult population. In the recently completed German Health Interview and Examination Survey for Adults (DEGS1, 2008-2011), self-reported as well as objectively measured health data related to chronic diseases, physical and cognitive functional capacities, and disability were collected. DEGS1 was designed to be nationally representative for German residents 18-79 years. Comparable data on several, but not all constructs and indicators are available from a previous national health survey for adults in Germany (German National Health Interview and Examination Survey 1998, GNHIES98).

For example, comparable information from both surveys was available for self-reported medical history of 15 chronic health conditions, subjective health and health-related quality of life as assessed by the *36-*Item *Short Form* Health Survey (*SF-36*), health-related behaviour, socioeconomic determinants, health care services utilisation, various anthropometric and biochemical measures, and verified information on current medication use. First analyses show that survey-weighted, age-standardised prevalence estimates of multi-morbidity (≥3 concurrent chronic conditions), obesity (body mass index ≥30 kg/m^2^), and poly-pharmacy (use of ≥4 prescription medications within 7 days prior to the survey) significantly increased in the population 65-79 years of age between 1998 and 2008-11; this was more pronounced among men than among women. Norm-based age standardised means of physical functioning (SF-36) improved over the same time, particularly among women.

These results underline that it is important to distinguish between the concepts of morbidity and disability when evaluating age-related changes in population health over time, as has been pointed out previously. Functional capacity represents a third multidimensional health concept as represented in the International Classification of Functioning, Disability and Health (ICF). Measures of physical and cognitive capabilities in the population 65-79 years have first been collected in DEGS1. Following DEGS1 study participants over time and adding on to the panel in future DEGS waves, opens the perspective to study health trajectories and to test hypothesized causal relationships.

